# Stage-specific transcriptomic changes in pancreatic α-cells after massive β-cell loss

**DOI:** 10.1186/s12864-021-07812-x

**Published:** 2021-08-02

**Authors:** Daniel Oropeza, Valentina Cigliola, Agustín Romero, Simona Chera, Santiago A. Rodríguez-Seguí, Pedro L. Herrera

**Affiliations:** 1 Department of Genetic Medicine and Development, iGE3 and Centre Facultaire du Diabète, Faculty of Medicine, University of Geneva, Geneva, Switzerland; 2 Present address: Department of Cell Biology, Duke University Medical Center, Durham, NC USA; 3 Regeneration Next, Duke University, Durham, North Carolina 27710 USA; 4 Departamento de Fisiología, Biología Molecular y Celular, Facultad de Ciencias Exactas y Naturales, Universidad de Buenos Aires and Instituto de Fisiología, Biología Molecular y Neurociencias (IFIBYNE), CONICET-Universidad de Buenos Aires, Ciudad Universitaria, Buenos Aires, Argentina; 5 Department of Clinical Science, Center for Diabetes Research, Faculty of Medicine, University of Bergen, Bergen, Norway

**Keywords:** Alpha cell, Beta cell, Pancreas, Pancreatic islet, Conversion, RNA-seq, Transcriptome, Plasticity, Regeneration, Ifit3

## Abstract

**Background:**

Loss of pancreatic insulin-secreting β-cells due to metabolic or autoimmune damage leads to the development of diabetes. The discovery that α-cells can be efficiently reprogrammed into insulin-secreting cells in mice and humans has opened promising avenues for innovative diabetes therapies. β-cell loss triggers spontaneous reprogramming of only 1–2% of α-cells, limiting the extent of regeneration. Most α-cells are refractory to conversion and their global transcriptomic response to severe β-cell loss as well as the mechanisms opposing their reprogramming into insulin producers are largely unknown. Here, we performed RNA-seq on FAC-sorted α-cells to characterize their global transcriptional responses at different time points after massive β-cell ablation.

**Results:**

Our results show that α-cells undergo stage-specific transcriptional changes 5- and 15-days post-diphtheria toxin (DT)-mediated β-cell ablation. At 5 days, α-cells transiently upregulate various genes associated with interferon signaling and proliferation, including Interferon Induced Protein with Tetratricopeptide Repeats 3 (*Ifit3*). Subsequently, at 15 days post β-cell ablation, α-cells undergo a transient downregulation of genes from several pathways including Insulin receptor, mTOR and MET signaling.

**Conclusions:**

The results presented here pinpoint novel markers discriminating α-cells at different stages after acute β-cell loss, and highlight additional signaling pathways that are modulated in α-cells in this context.

**Supplementary Information:**

The online version contains supplementary material available at 10.1186/s12864-021-07812-x.

## Background

Mounting data from multiple model organisms, spanning from hydra and axolotl to zebrafish and mammals, have revealed that injury triggers a strong and orchestrated transcriptional response in cells exposed to tissue damage and impacts not only inflammatory genes but also growth factors and signaling pathways controlling organ regeneration [1, 2]. Loss of pancreatic insulin-secreting β-cells due to metabolic or autoimmune damage leads to the development of diabetes. Following near-total β-cell loss in mice, 1–2% of α-cells stop glucagon production and spontaneously reprogram to insulin producers, as a direct consequence of insulin signaling deprivation [3–5]. Yet, most α-cells are refractory to conversion, limiting the extent of regeneration.

The discovery that α-cells can be efficiently reprogrammed into insulin-secreting cells in mice and, remarkably, in humans [6], has spurred intense research to dissect the underlying conversion mechanisms. Ectopic expression of the β-cell-specific transcription factors (TFs) Pdx1, Nkx6.1, Mafa or Pax4 can induce β-cell features in fetal or neonatal α-cells [7–10]. Conversely, inactivation of the α-cell-specific TF Arx and DNA methyltransferase 1, Dnmt1, in adult α-cells also promotes efficient conversion into insulin producers [11]. More recently, we showed that α-to-β-like cell conversion upon acute β-cell loss is constrained by intra-islet Smoothened and Insulin signaling. Smoothened-mediated Hedgehog signaling plays a key role in cell differentiation and maintenance in many organs, including pancreas [12–15], where it regulates *Pdx1* and *Ins* expression [16, 17]. Genetic inhibition of the Smoothened G protein-coupled receptor, together with β-cell loss enhances α-cell reprogramming [5]. Likewise, direct modulation of insulin signaling is sufficient to activate insulin expression in α-cells, without any associated β-cell death [5]. As well, ectopic expression of the TFs PDX1 and MAFA in human α-cells efficiently converts them into insulin-secreting cells that lead to diabetes reversal when transplanted into diabetic mice [6]. Taken together, these data suggest that various genetic mechanisms are dynamically integrated to regulate α-cell identity and plasticity in homeostatic or stress conditions. We have just begun to understand these complex α-cell genetic networks and crucial wide-ranging questions remain unanswered.

α-cell reprogramming only occurs in a subpopulation of α-cells. Yet, ≈98% of α-cells are refractory to conversion and do not transition towards a β-like cell fate. The global step-wise transcriptomic responses of most α-cells following severe β-cell loss, as well as the mechanisms opposing their reprogramming into insulin producers, are still unknown. To address this, and shed light on the mechanisms potentially restricting their reprogramming, we performed RNA-seq on FAC-sorted α-cells purified at different time points after near-total-β-cell ablation in mice. We confirmed the temporal modulation of Smoothened and Insulin signaling pathways, in agreement with our previous reports [5], and further identified novel pathways and putative markers of these reactive and transitional α-cell stages. One of the strongest responses is the early upregulation in α-cells of genes associated with Interferon signaling at 5 days post-β-cell ablation, which we confirm at the protein level for the Interferon Induced Protein with Tetratricopeptide Repeats 3 (*Ifit3*). These results expand our understanding of the genetic mechanisms underlying the α-cell response to β-cell loss and are expected to serve as a useful resource of putative α-cell stage-specific markers.

## Results

### Loss of β-cells triggers stage-specific global transcriptional changes in the α-cell population

**Fig. 1:**
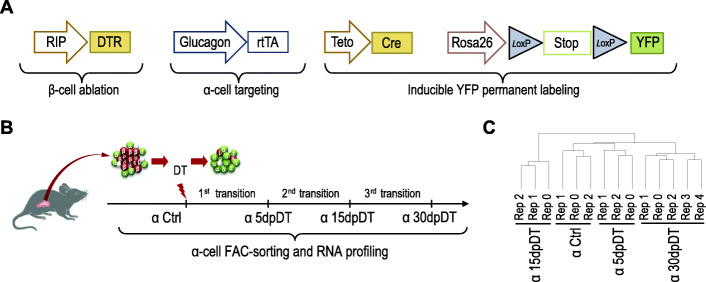
α-cells undergo stage-selective global transcriptional changes after diphtheria toxin (DT)-induced β-cell loss. **A** Schematic of the transgenic mice used in this work. **B** Experimental design. α-cell lineage tracing was performed administering doxycycline for 2 weeks in the drinking water. After 2 additional weeks of DOX clearance, β-cells were ablated by intraperitoneal diphtheria toxin injections on days 1, 3 and 5. α-cells were purified at 5, 15 and 30 days post-DT injection (dpDT). **C** Hierarchical clustering analysis shows that the transcriptomes from stage-specific α-cell samples present higher correlation with biological replicates than with samples from other stages

To begin dissecting the molecular programs dynamically regulated in α-cells following severe β-cell ablation, we FAC-sorted α-cells from *Glucagon-rtTA, TetO-Cre, R26-stop-YFP, RIP-DTR* transgenic mice (Fig. 1A). In these animals, doxycycline administration triggers irreversible labeling of α-cells with YFP, and diphtheria toxin (DT) injection causes > 99% β-cell death [3]. Mice were given subcutaneous insulin pellets 7 days after β-cell ablation when they were hyperglycemic (> 25 mM), as described [3, 5]. We validated that this approach stabilized the glycemia and ablated most β-cells at 15 days post-DT injection (dpDT, Figures S1A-S1C), as previously reported [3]. We performed bulk RNA-seq of α-cells at three time points: 5dpDT, 15dpDT and 30dpDT. These time points were chosen based on our previous observations, as follows: i) 5dpDT, to characterize α-cell early response to injury, ii) 15dpDT, the time when the first converted cells, still bi-hormonal, appear [3] and iii) 30dpDT, time when converted cells no longer expressing glucagon are present in the islets [3]. Native α-cells were used as controls, as previously published (Ctrl α-cells, Table S1, Fig. 1B) [5]. To assess the quality and reproducibility of the FAC-sorted α-cell replicates, we analyzed the Pearson correlation coefficient of pairwise-comparisons between gene expression profiles (14 samples in total: triplicates for Ctrl, 5dpDT and 15dpDT α-cells, and quintuplicates for 30dpDT α-cells), and performed unsupervised hierarchical clustering. Replicates clustered together, indicating reproducible differences in the gene expression profiles between the time points analyzed (Fig. 1C).

**Fig. 2:**
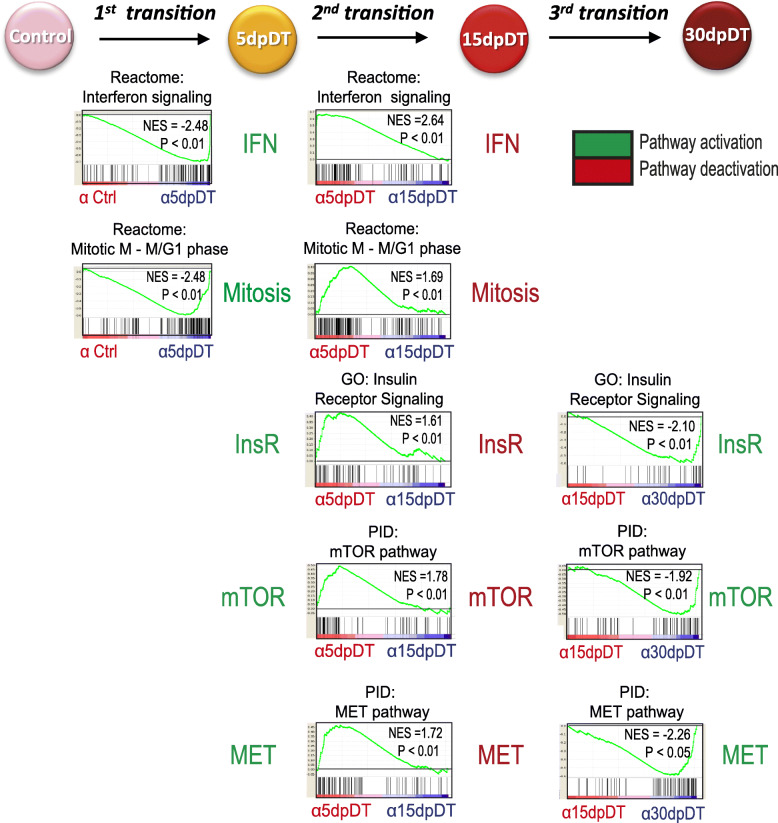
Analysis of the α-cell dynamic transcriptional response to β-cell loss reveals signaling pathways potentially modulating α-cell conversion towards insulin production. Selected Gene Set Enrichment Analysis (GSEA) results are presented (see also Figure S2). Genes with higher expression in 5dpDT α-cells, relative to its timely connected stages (Ctrl and 15dpDT α-cells) are associated with interferon signaling and control of the mitotic process. Genes downregulated in 15dpDT α-cells, relative to its timely connected stages (5dpDT and 30dpDT α-cells) are associated with inhibition of signaling from InsR, mTOR and MET pathways. All results presented are significant, considering a *P*-value < 0.05 and FDR < 0.25

To obtain an unbiased overview of the genetic pathways differentially modulated in α-cells after β-cell loss, we performed pairwise gene set enrichment analysis (GSEA) using the transcriptomes of timely connected α-cell stages (i.e. Ctrl→5dpDT; 5dpDT → 15dpDT and 15dpDT → 30dpDT transitions, Table S2). To capture subtle but collective significant gene expression changes in signaling pathways, we used all genes expressed in α-cells at each one of the stages analyzed. The initial transition (Ctrl→5dpDT) showed enrichment of genes related with inflammatory signaling and cell cycle regulation or cell replication in 5dpDT α-cells (Fig. 2**,** Table S2 in bold). This was transient, as the enrichment was not sustained in 15dpDT α-cells (Fig. 2). Interestingly, genes associated with “Smoothened signaling” were enriched in control α-cells, in agreement with our previous results showing that downregulation of Hedgehog signaling facilitates α-cell identity changes. In addition, genes belonging to the “Islet cell identity genes associated with enhancer clusters” category, encompassing 60 genes with well-documented roles in islet identity or function like *Rfx6*, *Pax6* and *glucokinase* [18] were also enriched in control compared to 5dpDT α-cells (Figure S2A, Table S2 in bold).

When focusing on the next α-cell transition (5dpDT → 15dpDT), GSEA again revealed enrichment of genes associated with cell proliferation and immune responses in 5dpDT α-cells but also Insulin receptor signaling, in agreement with our previous results showing that this signaling opposes α-cell reprogramming [5]. Other pathways showed a similar trend, including MET, TOR and Rac1 (Table S2, Fig. 2). Noteworthy, these pathways are associated with signaling mediated by the Rho family of GTPases RhoA, Rac1, and Cdc42, which are known to regulate actin cytoskeleton organization, direct cellular migration and polarity, and have been shown to play a role in pancreas organogenesis [19–22]. Genes associated with downregulation of Hedgehog signaling and with activation of Gαi signaling (shown to restrict islet cell expansion) [23] were enriched in 15dpDT α-cells, indicating that the initial proliferation attempt at 5dpDT might be blunted at this stage (Figure S2A, Table S2).

The last α-cell transition analyzed (15dpDT → 30dpDT) was characterized by regained expression of genes associated with Insulin receptor, mTOR, Rac1 and MET signaling in 30dpDT α-cells (Fig. 2, S2A). Thus, in contrast with findings from the previous transition, the transition to 30dpDT is characterized by increased signaling of pathways that were downregulated in 15dpDT α-cells. Taken together, the GSEA results point towards an initial and transient immune and proliferative α-cell response followed by a very dynamic activity in different signaling pathways characterized by a general transient downregulation at 15dpDT.

### α-Cells initiate sequential gene programs’ activation/deactivation after acute β-cell loss

**Fig. 3:**
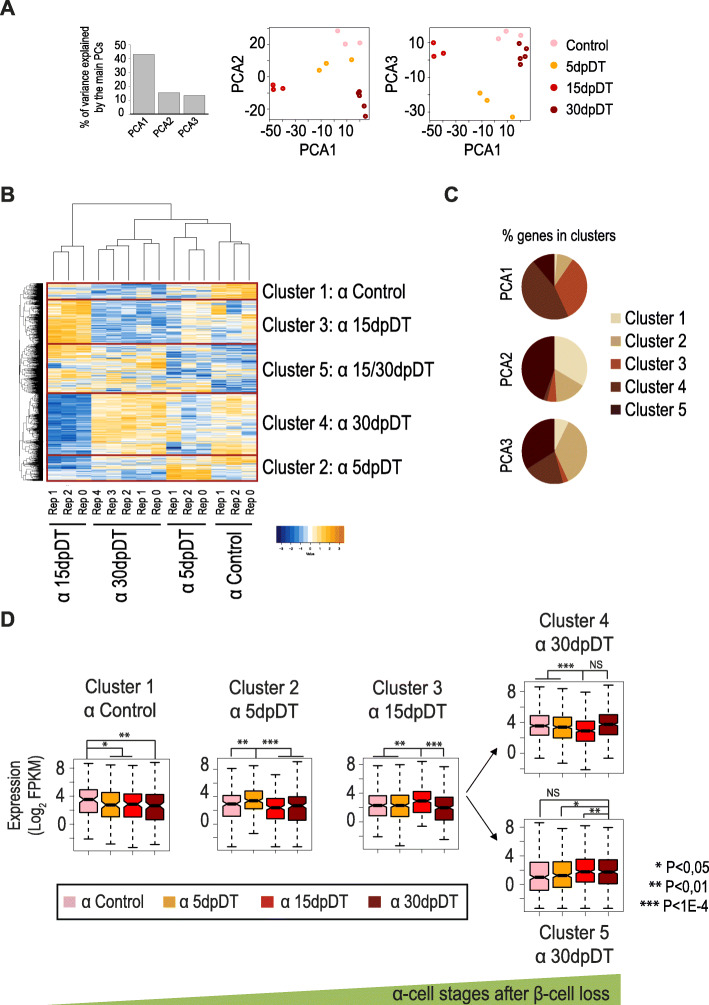
Key α-cell transitional gene expression signatures define α-cell stages after acute β-cell loss. **A** PCA shows that 1522 differentially expressed genes are enough to distinguish between α-cells from different stages after β-cell loss. The bar plot on the left depicts the percent of variance explained by each of the principal components (PC). PC1, accounting itself for 40% of the variance among samples, clearly separates α-cell 15dpDT replicates from the rest of the samples, suggesting that these cells present the most different transcriptomic signature. **B** Hierarchical clustering of 1522 differentially expressed genes identifies 5 gene clusters associated with specific α-cell signatures that are characteristic of each stage. **C** Clustered genes’ distribution across the PCA1, PCA2 and PCA3 dimensions. **D** The gene expression pattern characteristic of each cluster is associated with enrichment at specific α-cell stages. The boxes show the IQR of RNA levels, whiskers extend to 1.5 times the IQR or extreme values and notches indicate 95% confidence intervals of the median. The *P*-value was calculated with the Wilcoxon rank-sum test. NS: Not significant

We next sought to identify transcripts selectively enriched in α-cells that could serve as markers of specific time points after β-cell loss. For this purpose, we focused on the transcripts that showed significant changes in at least one of the pairwise comparisons performed between Ctrl, 5dpDT, 15dpDT and 30dpDT α-cells. After excluding genes considered not informative for the separation of stage-specific α-cell samples (see Methods, and Table S3) we retained a set of 1522 genes (Table S4). We herein refer to this set of genes as “α-cell transitional genes”. Indeed, Principal Component Analysis (with the first 3 components explaining more than 70% of the variance) revealed that “α-cell transitional genes” were sufficient to clearly cluster the replicates of each α-cell stage together, without any information provided a priori on the sample identity (Fig. 3A). Unsupervised hierarchical clustering analyses further separated α-cell transitional genes into 5 clusters with specific α-cell stage expression patterns after β-cell loss (see Methods, Fig. 3B, C, Table S4). Clusters 1 to 3 contained genes that were expressed at higher levels in either Ctrl, 5dpDT or 15dpDT α-cells, respectively (Fig. 3D). Genes from cluster 4 presented selective downregulation in 15dpDT α-cells, and recovered expression levels closer to Ctrl later in 30dpDT α-cells. Cluster 5 contained genes with a more heterogeneous pattern of expression, on average upregulated in 15dpDT α-cells, and then remaining highly expressed in 30dpDT α-cells (Fig. 3B, D).

These observations further suggest that after near total β-cell loss, the vast majority of α-cells initiate a sequential activation/deactivation of distinct gene programs. The clearest signature was detected in 15dpDT α-cells, with a strong upregulation of cluster 3 genes and downregulation of cluster 4 genes. Taken together, these 2 groups of genes account for 80% of the genes in PCA1 dimension (Fig. 3C), which clearly separated 15dpDT α-cells from the rest of the samples (Fig. 3A).

### Epigenomic analyses reveal stage-specific marker genes potentially driving the α-cell response to β-cell loss

**Fig. 4:**
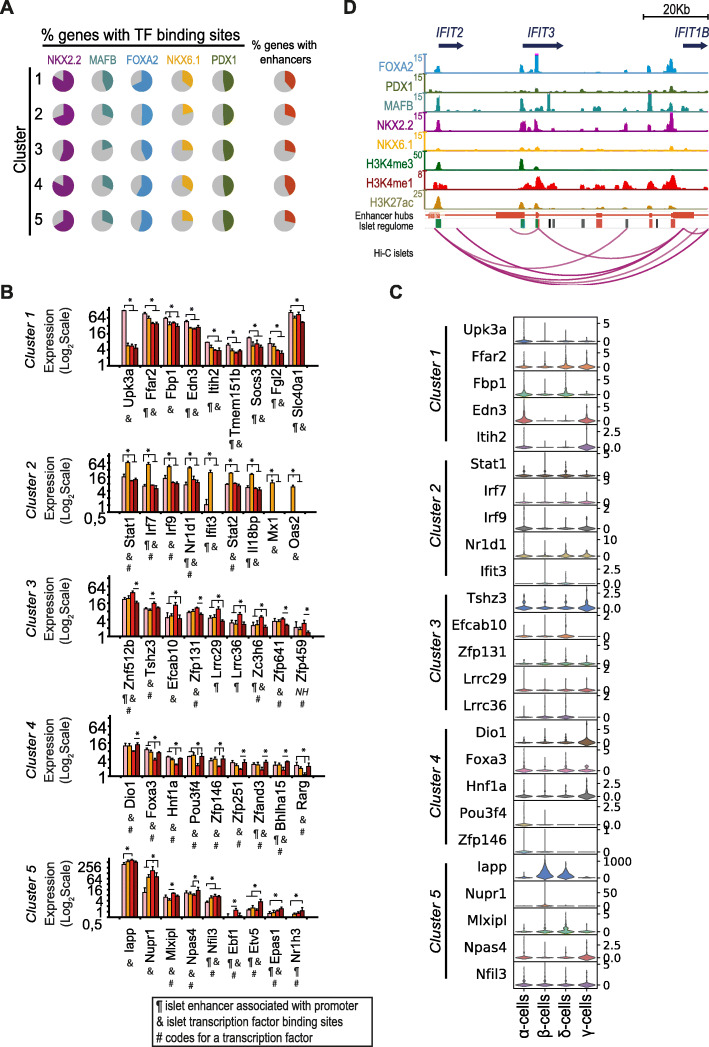
Epigenomic analyses reveal stage-specific marker genes potentially driving the α-cell response to β-cell loss. **A** Proportion of genes from each cluster associated with transcription factor binding sites, and proportion of cluster genes with their promoter interacting with at least 1 islet enhancer. **B** Expression profiles for selected cluster 1–5 markers. Codes below each gene name indicate association with at least one transcription factor binding site in human islets (&), promoter physical interaction (pcHi-C) with at least one human islet enhancer (¶) and whether the gene encodes for a transcription factor (#). NH: No human homolog found. * *P* < 0.05. **C** Violin plots showing the single-cell expression profiles for selected cluster marker genes in mouse pancreatic islet endocrine cell types (data from [28]). **D** Integrative map of the IFIT3 locus showing human islet ChIP-seq signal for 5 key pancreatic islet transcription factors, histone modification enrichment profiles associated with active (H3K27ac) promoter (H3K4me3) and enhancer (H3K4me1) regions, islet enhancer hubs, islet regulome regions and arcs representing high-confidence pcHi-C interactions in human islets (data from [18, 26])

We next sought to improve the genomic characterization of our predicted α-cell transitional genes. We and others have shown that islet-specific gene activity is controlled by islet transcription factor binding at gene promoters and regulatory regions often located hundreds of kilobases away [18, 24–26]. Under this rationale, we added a new layer of information to the α-cell transitional genes whereby their association with active or poised TF-bound regulatory regions in pancreatic islets reinforces their specific role in endocrine islet cell physiology. We thus annotated the number of pancreatic islet enhancers associated to the promoters of the α-cell transitional genes based on the recently published promoter capture Hi-C (pcHi-C) maps in human pancreatic islets [26]. This work refined existing human islet epigenome annotations by integrating ATAC-seq, ChIP-seq for proteins involved in chromatin looping (Mediator, cohesin and CTCF) and pcHi-C information to describe an accurate chromatin landscape in this tissue. Noteworthy, this comprised a large set of bona fide interacting regions at the genome-wide level, which was used to enhance the annotation of our α-cell transitional genes. To account for potential regulatory regions not captured in the pcHi-C maps, we also annotated the number of binding sites for 5 key islet transcription factors associated to gene promoters. These comprised binding sites for FOXA2, MAFB and NKX2.2, which are expressed in most islet cell types; the β-cell-specific factor NKX6.1; and the β/δ-cell-specific factor PDX1 (Table S4). Of note, despite it has been reported that the enhancer location per se can diverge between mice and humans, enhancers usually regulate gene expression within topologically associated domains (aka TADs). Indeed, TADs were shown to be highly conserved between mice and humans [27]. Thus, despite the actual genomic position of enhancers might differ, the number of enhancers physically interacting with gene promoters in humans is indicative of an active TAD in human islets, therefore suggesting that a similarly relevant regulation might take place in mice. This approach revealed that up to 40% of α-cell transitional genes had their promoters interacting with at least one enhancer in human islets based on pcHi-C maps. In addition, the majority of these genes presents nearby binding sites for one or more of the transcription factors profiled in human islets. Of note, a large fraction of genes (up to 80% of genes in clusters 1 and 4) was associated with NKX2.2 binding (Figs. 4A and S3A), consistent with an important role for this transcription factor in ensuring the α-cell fate [29]. We also noted that ~ 8% of the genes in each cluster code, as well, for transcription factors (Table S4). At the promoter level, out of the 1386 α-cell transitional genes with human homologs, 955 (68.9%) had accessible promoters in human islets (C1 elements from Pasquali et al. [18]), and 301 showed at least one TF binding event at these regions (Table S4).

Pancreatic islets are a heterogeneous tissue that is largely composed by β-cells, followed by α-cells and, to a lesser extent, δ- and γ-cells but also by resident immune cells as well as endothelial and neuronal cells. Thus, to further narrow our transitional gene set for markers enriched in islet endocrine cells, we analyzed published mouse single-cell RNA-seq data (Figure S3B) [28]. Combined with the epigenomic annotations described above, this approach allowed selecting α-cell transitional genes with preferential expression in mouse α- and β-cells and whose promoters associate with islet enhancers and/or with nearby binding sites for one or more key islet transcription factors. Selected examples from the 5 different clusters of α-cell transitional genes that meet these criteria are presented in Fig. 4B and C. Illustrative examples of the epigenomic profile in the proximity of some of the genes from each cluster are shown in Figs. 4D and S3A.

### Ifit3 expression is strongly induced in α-cells after extreme β-cell loss

**Fig. 5:**
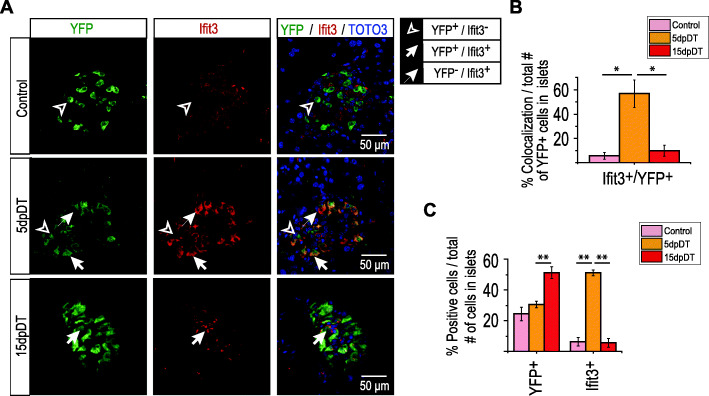
Ifit3 expression is strongly induced in α-cells after acute β-cell loss. **A** Representative immunofluorescence staining of pancreas sections obtained from Ctrl, 5dpDT and 15dpDT treated mice. **B** Percentage of cells co-expressing Ifit3 and YFP, expressed relative to the total number of total YFP+ cells in the islets. * *P* < 0.05. Total number of YFP+ cells (expressed as mean ± SEM) counted per replicate: Control (615 ± 268), 5dpDT (341 ± 184), 15dpDT (206 ± 48) from 3 biological replicates. **C** Percentage of cells expressing YFP, Ifit3, and co-expressing both markers, expressed relative to the total number of islet cells. Note that YFP+ cells represent the main cell type in 15dpDT islets given that by this timepoint β-cell ablation is almost complete. ** *P* < 0.01. Total number of islet cells (expressed as mean ± SEM) counted per replicate: Control (2291 ± 613), 5dpDT (1110 ± 455), 15dpDT (404 ± 96) from 3 biological replicates

An unexpected finding of our work is the strong and transient expression of genes associated with interferon signaling in 5dpDT α-cells. One of the most highly induced genes was the antiviral Interferon Induced Protein with Tetratricopeptide Repeats 3 (*Ifit3)* (Fig. 4B). To our knowledge, no reports have implicated *Ifit3* with any islet- or pancreatic-relevant physiological process. However, *Ifit3* presents several binding sites for the islet-specific transcription factors MAFB, FOXA2 and NKX2.2 at its promoter and enhancer-interacting regions (Fig. 4D). We thus sought to confirm whether *Ifit3* was induced in α-cells at the protein level after β-cell ablation. We used a previously reported [30], non-commercial, anti-Ifit3 antibody and performed immunofluorescence analysis in a new cohort of *Glucagon-rtTA, tetO-Cre, R26YFP, RIP-DTR* mice after inducing massive β-cell loss. To ensure specificity, we performed western blot analysis using mouse spleen extracts and showed the Ifit3 antibody recognized two bands matching the expected sizes for Ifit3 isoforms (Figure S3C). Ifit3 protein levels were highly and transiently induced 5dpDT in ~ 60% of YFP+ α-cells (Figs. 5A-B), while only ~ 10% YFP+ cells expressed Ifit3 in control and 15dpDT samples. Noteworthy, we also detected Ifit3 signal in other YFP- cells in the islet (Figs. 5A, C), including Insulin+ and Cd45+ immune cells (Figures S4, S5A). Moreover, Ifit3+ cells were also co-stained with glucagon, that at 5dpDT still closely mirrors YFP expression (Figure S5B). Additionally, in agreement with our GSEA results showing enrichment for categories involving immune system cell signaling interactions (e.g. “Antigen processing cross presentation” and “RIG I-like receptor signaling pathway”, among others, Table S2), we noticed a transient increase in the number of Cd45+ cells located in the islets at 5dpDT, in direct contact with YFP+ cells (both Ifit3+ and Ifit3-, Figures S4A-C). Extending our analysis to cover genes selectively induced in 15dpDT α-cells, we also validated the increased expression of Il2rg (a cluster 5 gene) at the protein level after β-cell ablation (Figure S6). Similarly to Ifit3, the role for this receptor in islet- or pancreatic-relevant physiological process remains largely unexplored.

## Discussion

Loss of β-cells triggers spontaneous reprogramming of a small α-cell population into insulin producers [3]. Yet, the global α-cell response to acute β-cell loss and the mechanisms opposing reprogramming of the vast majority of α-cells are still unknown. We describe here the global transcriptional changes of FAC-sorted mouse α-cells from 5 to 30 days after near-total β-cell loss, captured by 1522 “α-cell transitional genes”. Cross-referencing this gene set with a published human islet epigenomic landscape identified genes with islet-specific TF binding sites or transacting regulatory genomic regions, supporting an islet relevant functional role. This integrative analysis highlights potential α-cell markers of stage-specific responses to near-total β-cell loss, possibly involved in modulating α-cell conversion towards insulin producers. Noteworthy, here we aimed at characterizing the global α-cells’ response to loss of insulin-producing cells, hence we profiled them as a population. This approach prioritized capturing a larger number of genes with higher precision in expenses of detecting gene expression heterogeneity in the response of α-cells at the single cell level [31]. As such, our experimental approach does not allow to distinguish the α-cells profiled that will eventually convert at 30dpDT (only 1–2% of all α-cells), from those that will not reprogram (the remaining 98%). Thus, the transcriptomes profiled here are largely representative of cells refractory to conversion. Future single-cell RNA-seq studies are required to explore the transcriptome of reprogrammed α-cells only, which will shed light on the intermediate cell fates they undertake while progressing towards β-like cell fates.

Among others, *Uroplakin 3A* (*Upk3a*) and *Endothelin 3* (*Edn3*) showed sustained downregulation in α-cells as soon as 5dpDT. *Upk3a* is preferentially expressed in α-cells and *Edn3* in both α- and γ-cells (Figs. 4B-C, S3B). *Upk3a* could be a novel α-cell stress response gene, as it was upregulated two-fold in α-cells of diet-induced diabetic mice [32]. Interestingly, Edn3-like peptides have been patented for the treatment of metabolic disorders such as obesity and diabetes [33]. At 5dpDT, α-cells transiently upregulate various genes associated with proliferation. These changes occur in the majority of α-cells that do not undergo conversion. Due to the above-mentioned limitations in this study, we cannot discriminate how cells undergoing transdifferentiation behave in this context. At 5dpDT, α-cells also showed a transient and strong (up to 20-fold, Table S4) upregulation of immune response Stat and Irf transcription factors (*Stat1*, *Stat2*, *Irf7* and *Irf9*), and several interferon-stimulated genes (ISGs), including *Ifit1*, *Ifit2*, *Ifit3*, MX Dynamin Like GTPases 1 and 2 (*Mx1*, *Mx2*) and 2′-5′-Oligoadenylate Synthetases (*Oas1a*, *Oas1b*, *Oas1g*, *Oas2*, *Oasl1*, *Oasl2*). Many of these genes showed binding sites of islet-specific TFs in their promoters in human islets (Figure S3A). At 15dpDT, α-cells are characterized by a transient up- and down-regulation of a set of genes (clusters 3 and 4, Fig. 3B, Table S4), some of which code for specific TFs including *Znf512b*, *Tshz3* and *Zfp459* (Fig. 4B). We could not find any relevant information in the literature about the function of these TFs in pancreas or islets. Nonetheless, we could track several regulatory regions bound by islet-specific transcription factors which physically interact with their gene promoters (Table S4 and Figure S3A). Downregulated TFs (cluster 4) included *Foxa3*, *Hnf1a*, *Pou3f4*, *Zfp146*, *Zfp251*, *Zfand3* and *Bhlha15*. Several of these TFs have important functions in pancreas development and endocrine function including *Hnf1a* [34], and the α-cell markers *Pou3f4* and *Foxa3*, which control expression of the glucagon gene and have been used to promote ectopic α-cell features in conjunction with other TFs [35–37]. *Zfand3* has been previously associated with T2D susceptibility and is thought to be involved in the mechanism of insulin secretion [37–39]. Finally, 30dpDT α-cells are mainly characterized by a 2-fold increase in expression of the *Etv5* TF which has been implicated in controlling insulin secretion [40].

When looking at subtler but collectively significant gene expression changes, GSEA analyses showed decreased Smoothened and Insulin receptor signaling in 5dpDT and 15dpDT, respectively, in accordance with our previous data showing that inhibition of these pathways can drive insulin expression in α-cells [5]. The clear transcriptomic signature of 15dpDT α-cells was characterized by downregulation of signaling pathways putatively opposing the α-to-β-cell conversion (using Insulin receptor signaling as a proxy) which were reactivated in the 15dpDT → 30dpDT cell transition, including mTOR and Hepatocyte Growth Factor (HGF/c-MET). mTOR signaling is involved in islet cell differentiation [41] and its inhibition has been shown to promote islet cell reprogramming and β-cell regeneration [42]. Even though the Insulin receptor signaling is downregulated at 15dpDT, we cannot exclude a possible effect on α-cell gene expression of insulin administration at 7dpDT. Similarly, we cannot exclude direct effects of hyperglycemia or the influx of immune cells from the one attributed to acute β-cell loss. Simultaneous inhibition of multiple signaling pathways and systematic dissection of the effect of insulin on the α-cell transcriptome will be exciting areas for future research.

GSEA showed a robust and transient upregulation in 5dpDT α-cells of genes involved in several pathways of immune responses and cell cycle regulation. This is in accordance with various reports showing increased α-cell proliferation in mice shortly following β-cell ablation using chemical or transgenic methods [43–46] and even in α-cells from some Type 1 diabetics [47]. Thus, the transient upregulation of proliferative gene sets shows that our methodology is effective in detecting functionally relevant pathways. Upregulation of ISGs in 5dpDT α-cells is compatible with an initial pro-inflammatory environment triggered by infiltrating macrophages, characteristic of injure healing processes [48]. Indeed, complex immune cell interactions involving macrophages (as potentially indicated by Cd45+ cells in 5dpDT islet in Figure S4) have been implicated in tissue regeneration by modulating the transition from a pro-inflammatory to anti-inflammatory environment [49, 50]. Interestingly, the macrophage-associated factor HGF has been linked with i) induction of an anti-inflammatory macrophage phenotype [50], ii) liver regeneration [51], and iii) islet neogenesis and regeneration [50, 52].

In summary, our results further support a role of Smo and Insulin receptor signaling in modulating α-to-β-like cell conversion [5], and reveal other signaling pathways with similar enrichment patterns like mTOR and HGF/c-MET. We identify clear transcriptomic signatures at all α-cell stages analyzed, with modulation of expression of several transcription factors with unknown functions in α or other islet cells. Finally, we report that α-cell gene expression patterns reflecting interactions with immune cells are the strongest at 5dpDT but still present along all the α-cell transitions analyzed, and validate that Ifit3, a well-established ISG, is strongly induced at the protein level in 5dpDT α-cells.

## Conclusion

Our results show that α-cells undergo stage-specific transcriptional changes 5- and 15-days post-diphtheria toxin (DT)-mediated β-cell ablation, highlighting novel signaling pathways that are modulated in α-cells in this context. Combining our analysis with epigenomic and single-cell transcriptomic data of public domain further allowed us to pinpoint novel markers discriminating α-cells at different stages after acute β-cell loss. Taken together, these results expand our understanding of the genetic mechanisms potentially modulating α-cell response to β-cell loss, and are expected to serve as a useful resource of putative stage-specific α-cell markers.

## Materials and methods

### Mice

All transgenic mice were previously described [3]. Male mice were used for all experiments. This study is compliant with all relevant ethical regulations regarding animal research and all experiments have been approved and performed according to the guidelines of the Direction générale de la santé du Canton de Genève (license numbers: GE/103/14; GE/111/17 and GE/121/17).

### Doxycycline (DOX), diphtheria toxin (DT), and insulin treatments

α-cell lineage tracing was achieved administering doxycycline (DOX, Sigma) in the drinking water for 2 weeks, as previously described [3]. We waited 2 weeks to allow DOX clearance before any subsequent manipulation. To perform near-total β-cell ablation, three doses of DT (120 ng) were administered at days 1, 4 and 5 by intra-peritoneal (i.p.) injections as previously described [3]. Mice received a subcutaneous insulin pellet (Linbit) 1 week after the first DT injection when glycemia exceeded 25 mM.

### Islet isolation, FACS, RNA extraction and qPCR

Islet isolation, cell sorting, RNA preparation were performed as described [4].

Control α-cells and α-cells purified at 5, 15 and 30 days after DT (α 5dpDT, 15dpDT and 30dpDT) were sorted from *Glucagon-rtTA, R26YFP, RIP-DTR* mice (whose glucagon-expressing α-cells express the fluorescent reporter YFP and β-cells can be ablated by DT administration), injected with three doses of DT at 2 months of age.

### RNA-seq

RNA-seq was performed using ~ 20,000 cells per sample, pulled from 2 to 4 mice. Blood glucose was > 25 mmol/l at 5dpDT, as at this time point mice were not treated with insulin pellets. At 15 and 30dpDT blood glucose levels were < 25 mmol/l, as mice were under insulin treatment. RNA from cells isolated by FACS was assessed for quality by Agilent bioanalyzer prior to library generation and sequencing. Sequencing was performed at the iGE3 Genomic Platform using an HiSeq 2500 sequencer and we sequenced 100 bp single-end reads. To improve alignment rate, raw reads were trimmed to 60 bp using fastx_trimmer from the FASTX-Toolkit (http://hannonlab.cshl.edu/fastx_toolkit/). Trimmed reads were aligned with the Tuxedo suite (Bowtie2 2.2.2.0 [53], TopHat v2.0.12 [54], Cufflinks v2.2.1 [55]) using default settings and the UCSC mm10 annotation of the mouse genome, but changing the maximum number of fragments a locus may have before being skipped (−-max-bundle-frags) to 1E8, to include quantification of highly expressed α- and β-cell genes. See Table S1 for information about the number of total reads and the percentage of mapped reads for each sample. Transcript levels were quantified as fragments per kilobase of transcript per million mapped reads (FPKM) generated by TopHat/Cufflinks. Next, the Cuffnorm and Cuffdiff tools [55] were used to normalize gene expression data across samples and replicates used in this study and to detect differential gene expression in pairwise sample comparisons, respectively. Given that the sequencing depth of our samples (< 100 million reads) was not enough to accurately distinguish gene expression variants, for further analysis of gene expression we pooled expression data from splicing variants to have only one expression value per gene. Boxplots depicting expression of specific gene clusters (Fig. 3D) were performed using R [56].

Single-cell RNA-seq data from dissociated mouse pancreatic islets was taken from Baron et al. [28]. Already quantified and cell-type clustered data was downloaded from the Gene Expression Omnibus (GEO) with accession GSE84133. Data was processed for visualization of violin plots to show the expression of selected genes using the ScanPy package [57].

### ChIP-seq

Publicly available raw datasets were obtained from the Sequence Read Archive (SRA) database as listed in Table S1. Sequence reads were realigned to the human (UCSC hg19) genome using Bowtie v1.1.2 [53], and further processed as previously described [18, 58]. In brief, only sequences uniquely aligned with ≤1 mismatch were retained. Post-alignment processing of sequence reads included in silico extension and signal normalization based on the number of million mapped reads. Reads were extended to a final length equal to the MACS fragment size estimation [59], and only unique reads were retained. For signal normalization, the number of reads mapping to each base in the genome was counted using the genomeCoverageBed command from BedTools [60]. Processed files were visualized in the UCSC genome browser [61]. Transcription factor enrichment sites were detected with MACS v1.4.2 [59] using default parameters and a *P* value of 10^− 10^. We retained the peaks found in replicate experiments. We considered peaks to be overlapping if they shared a minimum of one base. Transcription factor binding sites were associated to the nearby genes using GREAT v4.0.4 with default settings [62]. Islet promoter regions were taken from Pasquali et al. [18] (C1 elements). These regions were lifted over from the hg18 to the hg19 genome annotation using the UCSC Genome Browser tool, and intersected with all annotated gene promoters (defined as windows spanning 1Kb upstream to 2Kb downstream from the gene transcription start sites) taken from the UCSC hg19 annotation. A similar approach was followed to identify TF binding events to annotated gene promoters. The α-cell transitional genes were further annotated based on these results (Table S4). The promoter capture Hi-C (pcHi-C) enhancer-gene promoter assignments in human pancreatic islets (Bait genes expressed in > 1.5 TPM in islets) were taken from Supplementary Data Set 2 of Miguel-Escalada et al. [26]. Screenshots for arcs representing high-confidence pcHi-C interactions in human islets were taken from the WashU Epigenome browser using this session link: http://epigenomegateway.wustl.edu/browser/?genome=hg19&session=62hGf7nfcS&statusId=140947077.

### Gene set enrichment analysis (GSEA)

GSEA v3.0 was performed using default parameters [63], but setting the gene set permutation type, a minimum geneset size to 50 genes and 2000 permutations, to increase the resolution of the results. The pairwise comparisons of the normalized transcriptome datasets obtained from FAC-sorted cells were analyzed using the canonical pathway geneset collection from the MsigDb database (c2.cp.v5.2.symbols.gmt) plus homemade genesets taken from recent literature reports [18, 64] or annotated in other MsigDb categories. Significantly enriched genesets (*p* < 0.05 and FDR < 0.25) in any of the pairwise comparisons are presented in Table S2.

### Clustering and principal component analyses (PCA)

The initial clustering of samples and replicates (Fig. 1C) was performed using all genes annotated in the UCSC (mm10 version). The Log2 expression values were scaled by the root mean square and Pearson’s correlation values were calculated for each pair. For the in-depth PCA and clustering of samples and transcripts (Fig. 3A, B) we used the pooled list of genes retrieved by Cuffdiff [55] as differentially expressed (up- or downregulated) in any of the α-cell sample pairwise comparisons considered (α Ctrl vs α 5dpDT, α 5dpDT vs α 15dpDT, α 15dpDT vs α 30dpDT, α Ctrl vs α 30dpDT, α Ctrl vs 15dpDT, α 5dpDT vs α 30dpDT). Based on the gene description of the UCSC mm10 genome annotation, we excluded from this list Ribosomal Protein genes, Pseudogenes, micro RNAs and Clone genes whose information was considered either not relevant due to the pleiotropic effect of these genes, or due to poor annotation and/or quantification (Table S3). An initial unsupervised hierarchical clustering analysis further identified a subset of acinar-associated genes that was significantly different among sample replicates and was associated with differences in the purity of the FAC-sorting protocol (Table S3). This set of genes was also excluded from the analysis. After processing, we finally kept a list of 1522 genes that were informative of the transcriptomic changes occurring in the α-cells at specific stages after β-cell ablation. The Log2 expression values were scaled by the root mean square. Clustering and PCA were performed using the heatmap.2 (gplots package) and PCA (FactoMineR package) functions in R [56], respectively.

### Homolog gene search

To find human homologs for mouse genes we used the Homologene database from the NCBI (available from https://www.ncbi.nlm.nih.gov/homologene). In cases were no homologs were found in this table, we further checked for gene homology annotations in the Gene database of the NCBI.

### Immunofluorescence

Immunolocalization studies were performed on paraffin sections of pancreas from *Glucagon-rtTA*; TetO-Cre; *R26-STOP-YFP*, *RIP-DTR* mice. Briefly, tissues were deparaffinated, re-hydrated and then were heated for 5 min for antigen retrieval. Sections were blocked on NDS (normal donkey serum) for 40 min and incubated with primary antibody for 24 h at 4 °C followed by incubation with secondary antibody overnight at 4 °C and nuclear staining with TOTO3 for 5 min.

The following primary antibodies were used in mice tissues: guinea pig anti-porcine insulin (DAKO, 1/400), mouse anti-glucagon (Sigma, 1/1000), mouse anti-somatostatin (BCBC Ab1985, 1/200) or goat anti-somatostatin (Santa Cruz 7918 1:200), chicken anti-GFP (Molecular Probes, 1/400), mouse anti-Il2rg (Santa Cruz sc-271,060 1:200). The rabbit-anti IFIT3 was a gift of Ganes Sen at Cleveland Clinic. This antibody recognized two bands matching the expected sizes for Ifit3 isoforms in a western blot validation analysis using protein extract from mouse spleen (Figure S3C). Secondary antibodies were coupled to Alexa 405, 488, 647, 547 (Molecular Probes). Sections were examined with a confocal microscope (Olympus FV-300).

### Statistical analyses

All mice used in experiments were males of mixed genetic background. Littermates with same genotype and age of experimental animals were used as controls. The number of mice per experiment was limited by the availability of required genotype and age. Criteria of exclusion were: inadequate transgene combination set, gender, evident signs of disease, including hyperglycemia before DT administration and spontaneous natural death during experiment.

Statistical analyses presented in Fig. 4B were taken from the Cuffdiff output. Gene expression q-values for comparison among samples within each barplot are summarized in Table S5.

Sample size for immunofluorescence analysis (scored numbers of mice islets and cells) is within the range of published literature in the field. Islets and cells were counted from multiple non-consecutive slides. All error bars represent s.e.m. = standard error of mean. *P*-values are given within figures or figure legends. ANOVA and Welch’s t-test were assessed using R as statistical software. For statistical analysis in Fig. 5B and C, One way ANOVA-Tukey’s HSD test and Welch’s t-test were assessed with R. For the quantification we used pancreases of three different 5dpDT and 15dpDT treated mice (*N* = 3) and 2 for Control mice (*N* = 2). Sample sizes, including the number of α-cells counted, used for statistic analysis presented in the figures showing immunofluorescence quantifications are summarized in Table S6.

## Supplementary Information

**Additional file 1: Figure S1.** Most insulin+ cells are ablated from 15dpDT and on. **(A)** Mean blood glucose levels for the mice used for the 5, 15 and 30 dpDT RNA-seq experiments. **(B)** Representative immunofluorescence staining of pancreas sections obtained from Control, 5dpDT and 15dpDT treated mice. **(C)** Percentage of cells expressing Insulin (Ins+) and YFP, expressed relative to the total number of cells in the islets. Total number of islet cells (expressed as mean ± SEM) counted per replicate: Control (784 ± 71, 3 biological replicates), 5dpDT (442 ± 111, 3 biological replicates), 15dpDT (301 ± 168, 2 biological replicates). **Figure S2.** Signaling pathways modulated in α-cells after acute β-cell loss. **(A)** Heatmap showing the Normalized Enrichment Score (NES) for selected Gene Set Enrichment Analysis (GSEA) results. T1: Ctrl vs 5dpDT α-cells, T2: 5dpDT vs 15dpDT α-cells, T3: 15dpDT vs 30dpDT α-cells. Genes with higher expression in Ctrl, relative to 5dpDT α-cells (i.e. enriched in Ctrl, with a positive NES), are associated with Smoothened signaling. Genes downregulated in 15dpDT, relative to 5dpDT α-cells (i.e. enriched in 5dpDT, with a positive NES), are associated with MET, mTOR and Rac1 signaling (among other pathways shown). Several of the pathways downregulated in T2 are upregulated in 30dpDT, relative to 15dpDT α-cells (see main text discussion for further details). All results presented are significant considering a *P*-value < 0.05 and FDR < 0.25. **(B)** Schematic summarizing the stepwise up- and downregulation of signaling pathways in α-cells as they transition from homeostasis (Ctrl α-cells) to 30dpDT. **Figure S3.** Epigenomic analysis reveals candidate genes and regulatory elements driving the α-cell response to β-cell loss. **(A)** Integrative epigenomic maps of the loci of selected cluster 1–5 marker genes showing human islet ChIP-seq signal for 5 key pancreatic islet transcription factors, histone modification enrichment profiles associated with active (H3K27ac) promoter (H3K4me3) and enhancer (H3K4me1) regions, islet enhancer hubs, islet regulome regions and arcs representing high-confidence pcHi-C interactions in human islets (data from (Pasquali et al. 2014 [18]) and (Miguel-Escalada et al. 2019 [26])). Note: OAS1 is the human homolog for Oas1g. **(B)** Violin plots showing the single-cell expression profiles for selected cluster marker genes in all the mouse pancreatic islet cell types identified by Baron et al. (Baron et al. 2016 [28])). **(C)** Western blot analysis for the anti-Ifit3 antibody using protein extract from mouse spleen. Gel image has been cropped to keep only relevant lanes. The molecular weight marker ladder and the Ifit3 western blot bands are part of the same image. Images have been taken using a LI-COR Odyssey equipment. Full-length blots/gels are presented as a supplementary file associated with Figure S3C. **Figure S4.** The presence of CD45+ cells is increased in islets after acute β-cell loss. **(A).** Immunofluorescence staining of pancreas sections obtained from Ctrl and 5dpDT treated mice. Control and 5dpDT bottom rows present zoom ins of regions indicated by white squares in the islets shown above. **(B).** Percentage of cells co-expressing Cd45 and YFP, expressed relative to the total number of total YFP+ cells in the islets. **(C).** Percentage of cells expressing Ifit3+ Cd45, and co-expressing both markers, expressed relative to the total number of Ifit3+ cells in the islets. Analysis focused on 5dpDT samples. Data for control samples is presented as reference. Values in panels **(B)** and **(C)** are expressed as the mean of 2 biological replicate experiments, with each value indicated by dots. Total number of islet cells counted per replicate: Control (replicate 1: 317, replicate 2: 76), 5dpDT (replicate 1: 368, replicate 2: 1236), 15dpDT (replicate 1: 126, replicate 2: 391). **Figure S5.** Ifit3 is also expressed in β-cells still present in 5dpDT islets. **(A).** Immunofluorescence images of pancreas sections obtained from Ctrl and 5dpDT treated mice showing Ifit3, Insulin and Glucagon co-staining. **(B).** Immunofluorescence images of pancreas sections obtained from 5dpDT treated mice showing YFP, Ifit3 and Glucagon co-staining. **Figure S6.** Il2r-g expression is strongly induced in α-cells after acute β-cell loss. **(A).** Representative immunofluorescence staining of pancreas sections obtained from Ctrl and 15dpDT treated mice. Note that Il2r-g is a protein mostly localized at the cell membrane, while YFP can present both nuclear and/or cytoplasmic stainings. Top right panels in 15dpDT images represent zoom ins of indicated regions. **(B).** Il2r-g gene expression in Control, 5dpDT, 15dpDT and 30dpDT as profiled by RNA-seq.

**Additional file 2: Supplementary Table 1.** Alignment details for raw RNA-seq and ChIP-seq data used in this study. **Supplementary Table 2.** Gene Set Enrichment Analysis significant (*p*<0.05, FDR<0.25) results obtained from pairwise sample comparisons. Categories mentioned in the manuscript are highlighted in bold. **Supplementary Table 3.** Genes excluded from the final clustering analysis. **Supplementary Table 4.** Lists of “α-cell transitional genes” including gene cluster association capturing the main differences between α-cells at different stages after β-cell loss. Number of human pancreatic islet enhancers, and number islet transcription binding sites for five islet transcription factors, that were associated with the promoters of genes in clusters 1 to 5. **Supplementary Table 5.** Statistical tests for genes showed in Figure 4B. **Supplementary Table 6.** Number of cells counted for the quantifications presented in the manuscript figures.

## Data Availability

All raw and processed sequencing data generated in this study have been submitted to the NCBI’s Gene Expression Omnibus (GEO; https://www.ncbi.nlm.nih.gov/geo/) under accession number **GSE155519**.
