# Supplementary material for: Stage-specific transcriptomic changes in pancreatic α-cells after massive β-cell loss
Source: BMC Genomics. 2021 Aug 2;22:585. doi: 10.1186/s12864-021-07812-x (PMC8330016; doi:10.1186/s12864-021-07812-x)
Supplement: Supplementary file 1 — Additional file 1: Figure S1. Most insulin+ cells are ablated from 15dpDT and on. (A) Mean blood glucose levels for the mice used for the 5, 15 and 30 dpDT RNA-seq experiments. (B) Representative immunofluorescence staining of pancreas sections obtained from Control, 5dpDT and 15dpDT treated mice. (C) Percentage of cells expressing Insulin (Ins+) and YFP, expressed relative to the total number of cells in the islets. Total number of islet cells (expressed as mean ± SEM) counted per replicate: Control (784 ± 71, 3 biological replicates), 5dpDT (442 ± 111, 3 biological replicates), 15dpDT (301 ± 168, 2 biological replicates). Figure S2. Signaling pathways modulated in α-cells after acute β-cell loss. (A) Heatmap showing the Normalized Enrichment Score (NES) for selected Gene Set Enrichment Analysis (GSEA) results. T1: Ctrl vs 5dpDT α-cells, T2: 5dpDT vs 15dpDT α-cells, T3: 15dpDT vs 30dpDT α-cells. Genes with higher expression in Ctrl, relative to 5dpDT α-cells (i.e. enriched in Ctrl, with a positive NES), are associated with Smoothened signaling. Genes downregulated in 15dpDT, relative to 5dpDT α-cells (i.e. enriched in 5dpDT, with a positive NES), are associated with MET, mTOR and Rac1 signaling (among other pathways shown). Several of the pathways downregulated in T2 are upregulated in 30dpDT, relative to 15dpDT α-cells (see main text discussion for further details). All results presented are significant considering a P-value < 0.05 and FDR < 0.25. (B) Schematic summarizing the stepwise up- and downregulation of signaling pathways in α-cells as they transition from homeostasis (Ctrl α-cells) to 30dpDT. Figure S3. Epigenomic analysis reveals candidate genes and regulatory elements driving the α-cell response to β-cell loss. (A) Integrative epigenomic maps of the loci of selected cluster 1–5 marker genes showing human islet ChIP-seq signal for 5 key pancreatic islet transcription factors, histone modification enrichment profiles associated with active (H3K27ac) promo [file 12864_2021_7812_MOESM1_ESM.zip › Oropeza_et_al_Suppl_Fig_Captions_Review_Round4.docx]

**Stage-specific transcriptomic changes in pancreatic α-cells**

**after massive β-cell loss**

**By**

Daniel Oropeza^1*^, Valentina Cigliola^1,2,3*^, Agustín Romero^4*^, Simona Chera^1,5^

Santiago A. Rodríguez-Seguí^4,6,ϕ^; Pedro L. Herrera^1,ϕ^

^1^ Department of Genetic Medicine and Development, iGE3 and Centre facultaire du diabète, Faculty of Medicine, University of Geneva, Geneva, Switzerland.

^2^ Present address: Department of Cell Biology, Duke University Medical Center, Durham, NC, USA.

^3^ Regeneration Next, Duke University, Durham, North Carolina, 27710, USA

^4^ Instituto de Fisiología, Biología Molecular y Neurociencias (IFIBYNE-UBA-CONICET), Universidad de Buenos Aires, Facultad de Ciencias Exactas y Naturales, Buenos Aires, [C1428EGA], Argentina.

^5^ Department of Clinical Science, Center for Diabetes Research, Faculty of Medicine, University of Bergen, Bergen, Norway

^6^ Departamento de Fisiología, Biología Molecular y Celular, Universidad de Buenos Aires, Facultad de Ciencias Exactas y Naturales, Ciudad Universitaria, Buenos Aires, [C1428EGA], Argentina.

^*^ Equal contribution.

^ϕ^ Correspondence should be addressed to S.A.R-S. or P.L.H. [srodriguez@fbmc.fcen.uba.ar](mailto:srodriguez@fbmc.fcen.uba.ar), [pedro.herrera@unige.ch](mailto:pedro.herrera@unige.ch).

**Running title: : α-cell transcriptional profile upon β-cell loss**

**Supplementary Figure Captions**

***Figure S1.*** *Most insulin+ cells are ablated from 15dpDT and on.* ***(A)*** *Mean blood glucose levels for the mice used for the 5, 15 and 30 dpDT RNA-seq experiments.* ***(B)*** *Representative immunofluorescence staining of pancreas sections obtained from Control, 5dpDT and 15dpDT treated mice.* ***(C)*** *Percentage of cells expressing Insulin (Ins+) and YFP, expressed relative to the total number of cells in the islets. Total number of islet cells (expressed as mean ± SEM) counted per replicate: Control (784 ± 71, 3 biological replicates), 5dpDT (442 ± 111, 3 biological replicates), 15dpDT (301 ± 168, 2 biological replicates).*

***Figure S2.*** *Signaling pathways modulated in α-cells after acute* β*-cell loss.* ***(A)*** *Heatmap showing the Normalized Enrichment Score (NES) for selected Gene Set Enrichment Analysis (GSEA) results. T1: Ctrl vs 5dpDT α-cells, T2: 5dpDT vs 15dpDT α-cells , T3: 15dpDT vs 30dpDT α-cells. Genes with higher expression in Ctrl, relative to 5dpDT α-cells (i.e. enriched in Ctrl, with a positive NES), are associated with Smoothened signaling. Genes downregulated in 15dpDT, relative to 5dpDT α-cells (i.e. enriched in 5dpDT, with a positive NES), are associated with MET, mTOR and RAC1 signaling (among other pathways shown). Several of the pathways downregulated in T2 are upregulated in 30dpDT, relative to 15dpDT α-cells (see main text discussion for further details). All results presented are significant considering a P-value<0.05 and FDR<0.25.* ***(B)*** *Schematic summarizing the stepwise up- and downregulation of signaling pathways in α-cells as they transition from homeostasis (Ctrl α-cells) to 30dpDT.*

***Figure S3.*** *Epigenomic analysis reveals candidate genes and regulatory elements driving the α-cell response to* β*-cell loss.* ***(A)*** *Integrative epigenomic maps of the loci of selected cluster 1-5 marker genes showing human islet ChIP-seq signal for 5 key pancreatic islet transcription factors, histone modification enrichment profiles associated with active (H3K27ac) promoter (H3K4me3) and enhancer (H3K4me1) regions, islet enhancer hubs, islet regulome regions and arcs representing high-confidence pcHi-C interactions in human islets (data from (Pasquali et al. 2014) and (Miguel-Escalada et al. 2019)). Note: OAS1 is the human homolog for Oas1g.* ***(B)*** *Violin plots showing the single-cell expression profiles for selected cluster marker genes in all the mouse pancreatic islet cell types identified by Baron et al. (Baron et al. 2016)).* ***(C)*** *Western blot analysis for the anti-Ifit3 antibody using protein extract from mouse spleen. Gel image has been cropped to keep only relevant lanes. The molecular weight marker ladder and the Ifit3 western blot bands are part of the same image.* *Images have been taken using a LI-COR Odyssey equipment. Full-length blots/gels are presented as a supplementary file associated with Figure S3C.*

***Figure S4.*** *The* *presence of CD45+ cells* *is increased in islets after acute* β*-cell loss.* ***(A).*** *Immunofluorescence staining of pancreas sections obtained from Ctrl and 5dpDT treated mice. Control and 5dpDT bottom rows present zoom ins of regions indicated by white squares in the islets shown above.* ***(B).*** *Percentage of cells co-expressing Cd45 and YFP, expressed relative to the total number of total YFP+ cells in the islets.* ***(C).*** *Percentage of cells expressing Ifit3+ Cd45, and co-expressing both markers, expressed relative to the total number of Ifit3+ cells in the islets. Analysis focused on 5dpDT samples. Data for control samples is presented as reference. Values in panels* ***(B)*** *and* ***(C)*** *are expressed as the mean of 2 biological replicate experiments, with each value indicated by dots. Total number of islet cells counted per replicate: Control (replicate 1: 317, replicate 2: 76), 5dpDT (replicate 1: 368, replicate 2: 1236), 15dpDT (replicate 1: 126, replicate 2: 391).*

***Figure S5.*** *Ifit3 is also expressed in* β*-cells still present in 5dpDT islets.* ***(A).*** *Immunofluorescence images of pancreas sections obtained from Ctrl and 5dpDT treated mice showing Ifit3, Insulin and Glucagon co-staining.* ***(B).*** *Immunofluorescence images of pancreas sections obtained from 5dpDT treated mice showing YFP, Ifit3 and Glucagon co-staining.*

***Figure S6.*** *Il2r-g expression is strongly induced in α-cells after acute* β*-cell loss.* ***(A).*** *Representative immunofluorescence staining of pancreas sections obtained from Ctrl and 15dpDT treated mice. Note that Il2r-g is a protein mostly localized at the cell membrane, while YFP can present both nuclear and/or cytoplasmic stainings. Top right panels in 15dpDT images represent zoom ins of indicated regions.* ***(B).*** *Il2r-g gene expression in Control, 5dpDT, 15dpDT and 30dpDT as profiled by RNA-seq.*
