# Supplementary figures and images for: Stage-specific transcriptomic changes in pancreatic α-cells after massive β-cell loss
Source: BMC Genomics. 2021 Aug 2;22:585. doi: 10.1186/s12864-021-07812-x (PMC8330016; doi:10.1186/s12864-021-07812-x)

**A**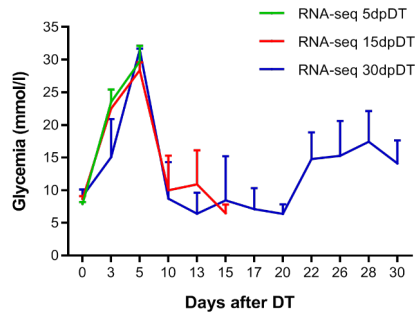**C**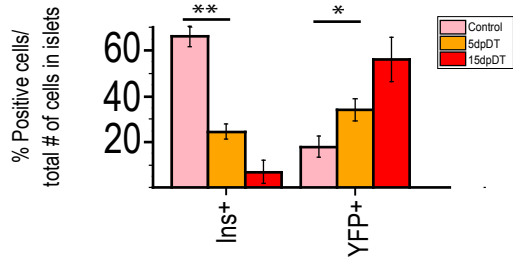**B**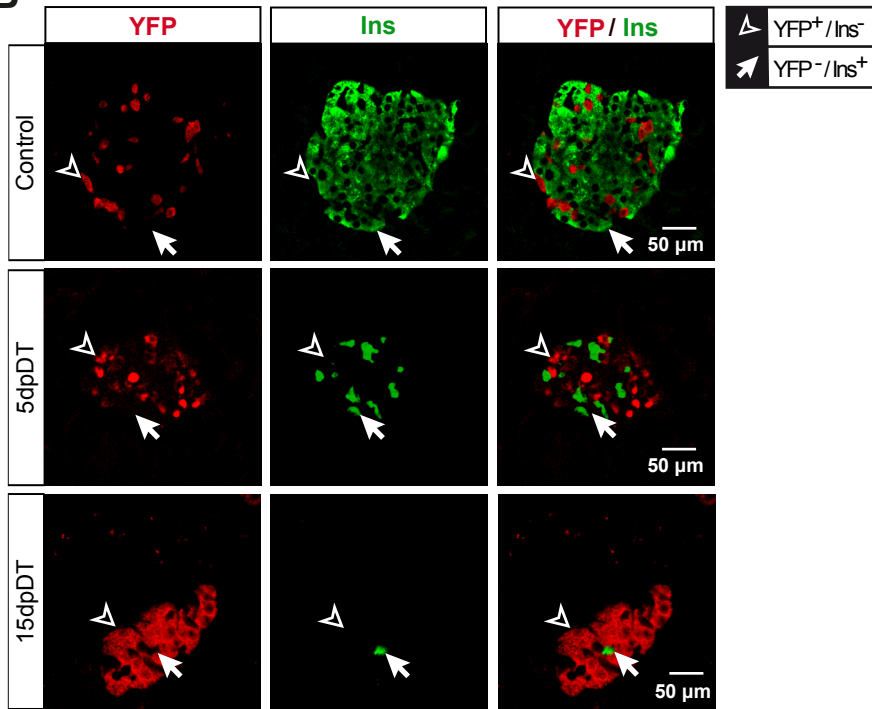

Supplement: Supplementary file 1 — Additional file 1: Figure S1. Most insulin+ cells are ablated from 15dpDT and on. (A) Mean blood glucose levels for the mice used for the 5, 15 and 30 dpDT RNA-seq experiments. (B) Representative immunofluorescence staining of pancreas sections obtained from Control, 5dpDT and 15dpDT treated mice. (C) Percentage of cells expressing Insulin (Ins+) and YFP, expressed relative to the total number of cells in the islets. Total number of islet cells (expressed as mean ± SEM) counted per replicate: Control (784 ± 71, 3 biological replicates), 5dpDT (442 ± 111, 3 biological replicates), 15dpDT (301 ± 168, 2 biological replicates). Figure S2. Signaling pathways modulated in α-cells after acute β-cell loss. (A) Heatmap showing the Normalized Enrichment Score (NES) for selected Gene Set Enrichment Analysis (GSEA) results. T1: Ctrl vs 5dpDT α-cells, T2: 5dpDT vs 15dpDT α-cells, T3: 15dpDT vs 30dpDT α-cells. Genes with higher expression in Ctrl, relative to 5dpDT α-cells (i.e. enriched in Ctrl, with a positive NES), are associated with Smoothened signaling. Genes downregulated in 15dpDT, relative to 5dpDT α-cells (i.e. enriched in 5dpDT, with a positive NES), are associated with MET, mTOR and Rac1 signaling (among other pathways shown). Several of the pathways downregulated in T2 are upregulated in 30dpDT, relative to 15dpDT α-cells (see main text discussion for further details). All results presented are significant considering a P-value < 0.05 and FDR < 0.25. (B) Schematic summarizing the stepwise up- and downregulation of signaling pathways in α-cells as they transition from homeostasis (Ctrl α-cells) to 30dpDT. Figure S3. Epigenomic analysis reveals candidate genes and regulatory elements driving the α-cell response to β-cell loss. (A) Integrative epigenomic maps of the loci of selected cluster 1–5 marker genes showing human islet ChIP-seq signal for 5 key pancreatic islet transcription factors, histone modification enrichment profiles associated with active (H3K27ac) promo [file 12864_2021_7812_MOESM1_ESM.zip › Figure_S1_new.pdf]

**A**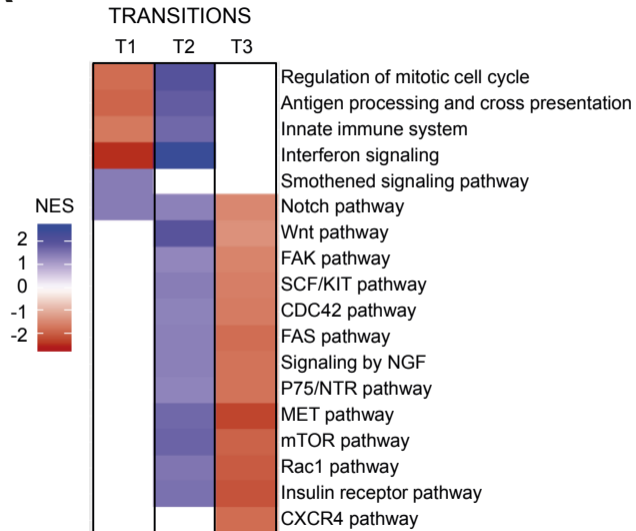**B**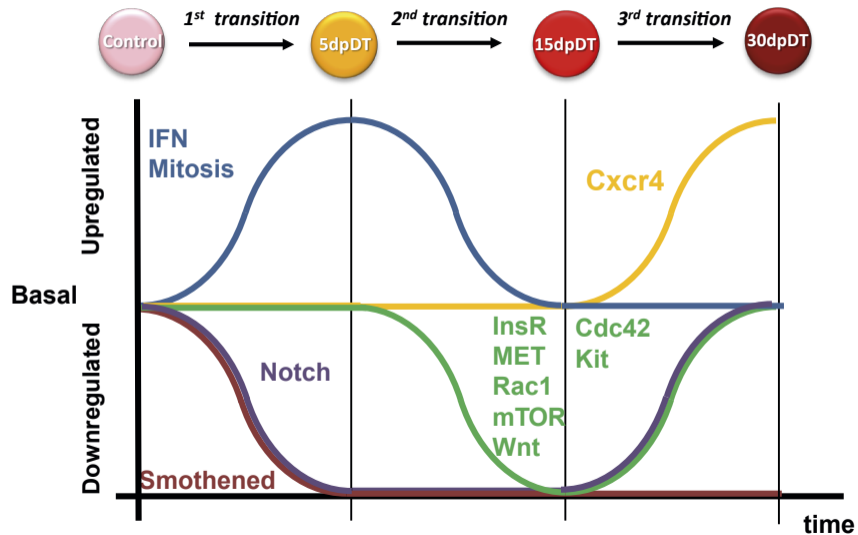

Supplement: Supplementary file 1 — Additional file 1: Figure S1. Most insulin+ cells are ablated from 15dpDT and on. (A) Mean blood glucose levels for the mice used for the 5, 15 and 30 dpDT RNA-seq experiments. (B) Representative immunofluorescence staining of pancreas sections obtained from Control, 5dpDT and 15dpDT treated mice. (C) Percentage of cells expressing Insulin (Ins+) and YFP, expressed relative to the total number of cells in the islets. Total number of islet cells (expressed as mean ± SEM) counted per replicate: Control (784 ± 71, 3 biological replicates), 5dpDT (442 ± 111, 3 biological replicates), 15dpDT (301 ± 168, 2 biological replicates). Figure S2. Signaling pathways modulated in α-cells after acute β-cell loss. (A) Heatmap showing the Normalized Enrichment Score (NES) for selected Gene Set Enrichment Analysis (GSEA) results. T1: Ctrl vs 5dpDT α-cells, T2: 5dpDT vs 15dpDT α-cells, T3: 15dpDT vs 30dpDT α-cells. Genes with higher expression in Ctrl, relative to 5dpDT α-cells (i.e. enriched in Ctrl, with a positive NES), are associated with Smoothened signaling. Genes downregulated in 15dpDT, relative to 5dpDT α-cells (i.e. enriched in 5dpDT, with a positive NES), are associated with MET, mTOR and Rac1 signaling (among other pathways shown). Several of the pathways downregulated in T2 are upregulated in 30dpDT, relative to 15dpDT α-cells (see main text discussion for further details). All results presented are significant considering a P-value < 0.05 and FDR < 0.25. (B) Schematic summarizing the stepwise up- and downregulation of signaling pathways in α-cells as they transition from homeostasis (Ctrl α-cells) to 30dpDT. Figure S3. Epigenomic analysis reveals candidate genes and regulatory elements driving the α-cell response to β-cell loss. (A) Integrative epigenomic maps of the loci of selected cluster 1–5 marker genes showing human islet ChIP-seq signal for 5 key pancreatic islet transcription factors, histone modification enrichment profiles associated with active (H3K27ac) promo [file 12864_2021_7812_MOESM1_ESM.zip › Figure_S2_new.pdf]

**A**

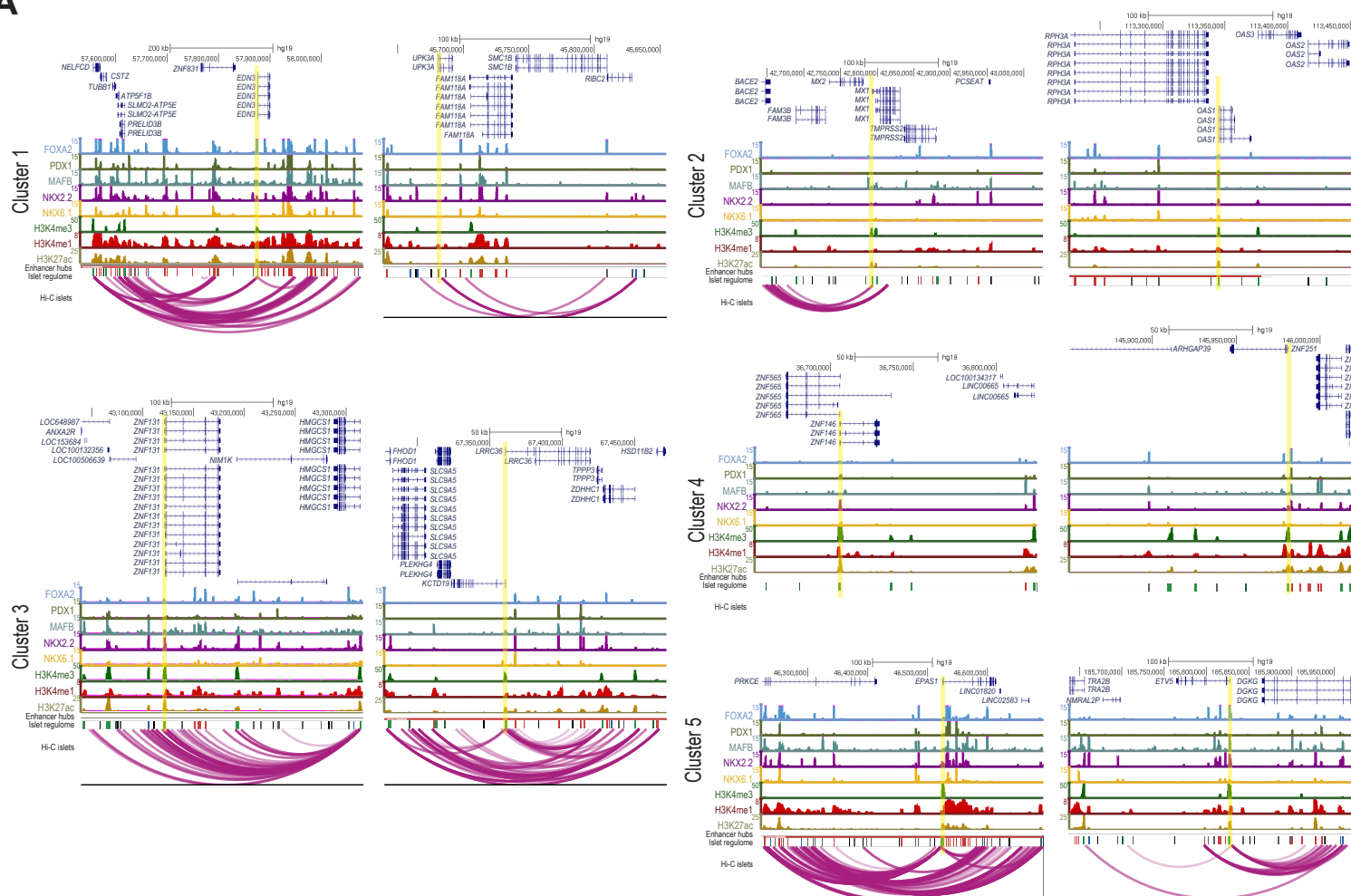

**B**

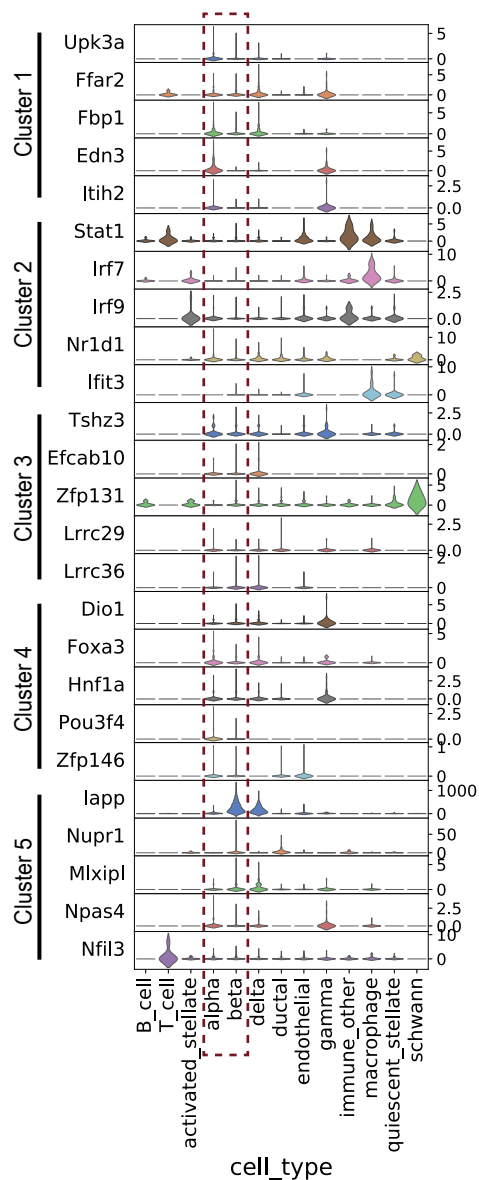

**C**

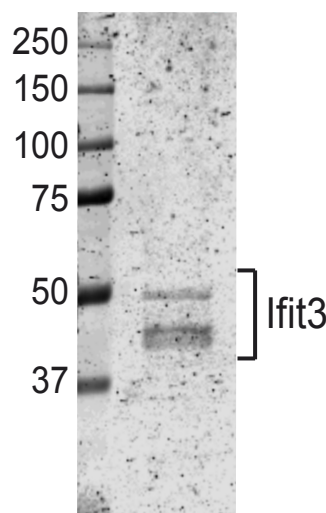

Supplement: Supplementary file 1 — Additional file 1: Figure S1. Most insulin+ cells are ablated from 15dpDT and on. (A) Mean blood glucose levels for the mice used for the 5, 15 and 30 dpDT RNA-seq experiments. (B) Representative immunofluorescence staining of pancreas sections obtained from Control, 5dpDT and 15dpDT treated mice. (C) Percentage of cells expressing Insulin (Ins+) and YFP, expressed relative to the total number of cells in the islets. Total number of islet cells (expressed as mean ± SEM) counted per replicate: Control (784 ± 71, 3 biological replicates), 5dpDT (442 ± 111, 3 biological replicates), 15dpDT (301 ± 168, 2 biological replicates). Figure S2. Signaling pathways modulated in α-cells after acute β-cell loss. (A) Heatmap showing the Normalized Enrichment Score (NES) for selected Gene Set Enrichment Analysis (GSEA) results. T1: Ctrl vs 5dpDT α-cells, T2: 5dpDT vs 15dpDT α-cells, T3: 15dpDT vs 30dpDT α-cells. Genes with higher expression in Ctrl, relative to 5dpDT α-cells (i.e. enriched in Ctrl, with a positive NES), are associated with Smoothened signaling. Genes downregulated in 15dpDT, relative to 5dpDT α-cells (i.e. enriched in 5dpDT, with a positive NES), are associated with MET, mTOR and Rac1 signaling (among other pathways shown). Several of the pathways downregulated in T2 are upregulated in 30dpDT, relative to 15dpDT α-cells (see main text discussion for further details). All results presented are significant considering a P-value < 0.05 and FDR < 0.25. (B) Schematic summarizing the stepwise up- and downregulation of signaling pathways in α-cells as they transition from homeostasis (Ctrl α-cells) to 30dpDT. Figure S3. Epigenomic analysis reveals candidate genes and regulatory elements driving the α-cell response to β-cell loss. (A) Integrative epigenomic maps of the loci of selected cluster 1–5 marker genes showing human islet ChIP-seq signal for 5 key pancreatic islet transcription factors, histone modification enrichment profiles associated with active (H3K27ac) promo [file 12864_2021_7812_MOESM1_ESM.zip › Figure_S3.pdf]

**A**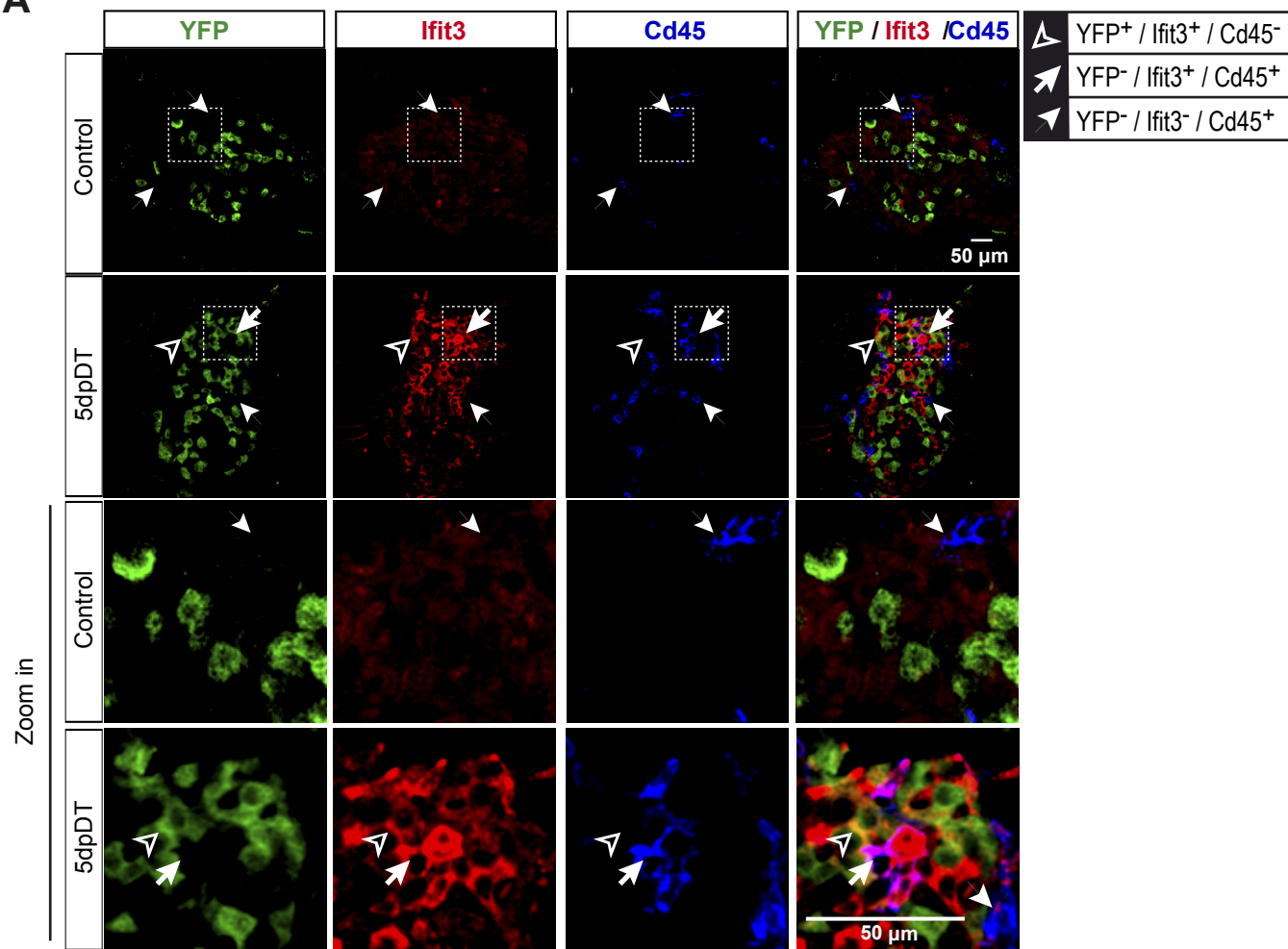**B**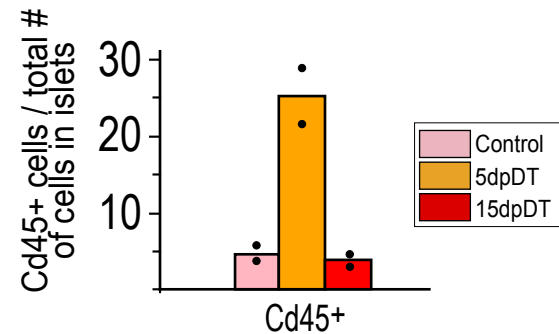**C**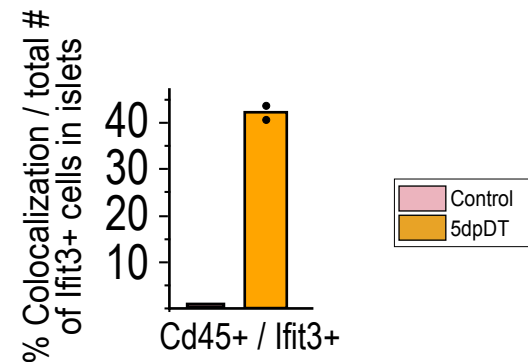

Supplement: Supplementary file 1 — Additional file 1: Figure S1. Most insulin+ cells are ablated from 15dpDT and on. (A) Mean blood glucose levels for the mice used for the 5, 15 and 30 dpDT RNA-seq experiments. (B) Representative immunofluorescence staining of pancreas sections obtained from Control, 5dpDT and 15dpDT treated mice. (C) Percentage of cells expressing Insulin (Ins+) and YFP, expressed relative to the total number of cells in the islets. Total number of islet cells (expressed as mean ± SEM) counted per replicate: Control (784 ± 71, 3 biological replicates), 5dpDT (442 ± 111, 3 biological replicates), 15dpDT (301 ± 168, 2 biological replicates). Figure S2. Signaling pathways modulated in α-cells after acute β-cell loss. (A) Heatmap showing the Normalized Enrichment Score (NES) for selected Gene Set Enrichment Analysis (GSEA) results. T1: Ctrl vs 5dpDT α-cells, T2: 5dpDT vs 15dpDT α-cells, T3: 15dpDT vs 30dpDT α-cells. Genes with higher expression in Ctrl, relative to 5dpDT α-cells (i.e. enriched in Ctrl, with a positive NES), are associated with Smoothened signaling. Genes downregulated in 15dpDT, relative to 5dpDT α-cells (i.e. enriched in 5dpDT, with a positive NES), are associated with MET, mTOR and Rac1 signaling (among other pathways shown). Several of the pathways downregulated in T2 are upregulated in 30dpDT, relative to 15dpDT α-cells (see main text discussion for further details). All results presented are significant considering a P-value < 0.05 and FDR < 0.25. (B) Schematic summarizing the stepwise up- and downregulation of signaling pathways in α-cells as they transition from homeostasis (Ctrl α-cells) to 30dpDT. Figure S3. Epigenomic analysis reveals candidate genes and regulatory elements driving the α-cell response to β-cell loss. (A) Integrative epigenomic maps of the loci of selected cluster 1–5 marker genes showing human islet ChIP-seq signal for 5 key pancreatic islet transcription factors, histone modification enrichment profiles associated with active (H3K27ac) promo [file 12864_2021_7812_MOESM1_ESM.zip › Figure_S4_Review_Round3.pdf]

**A**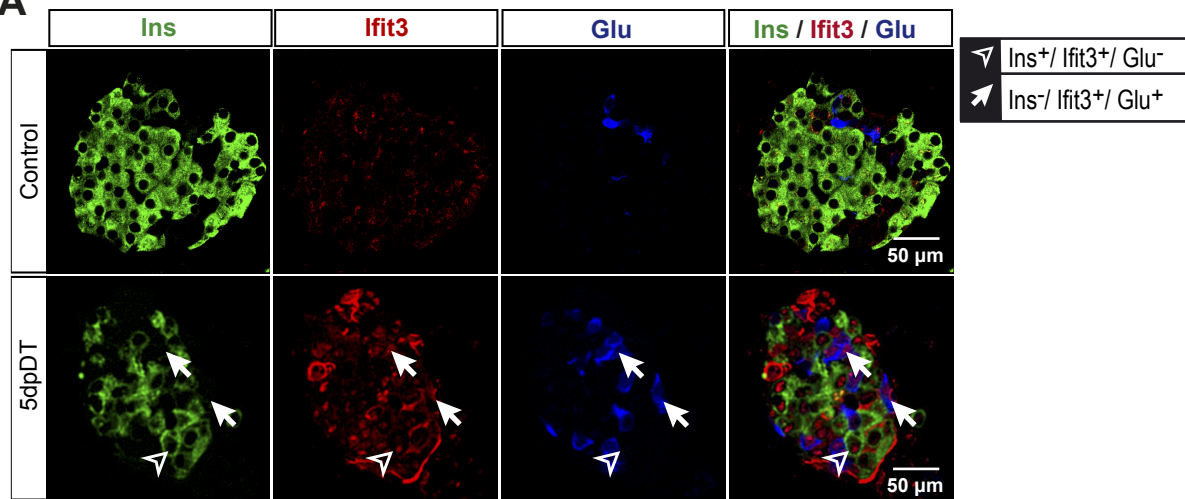**B**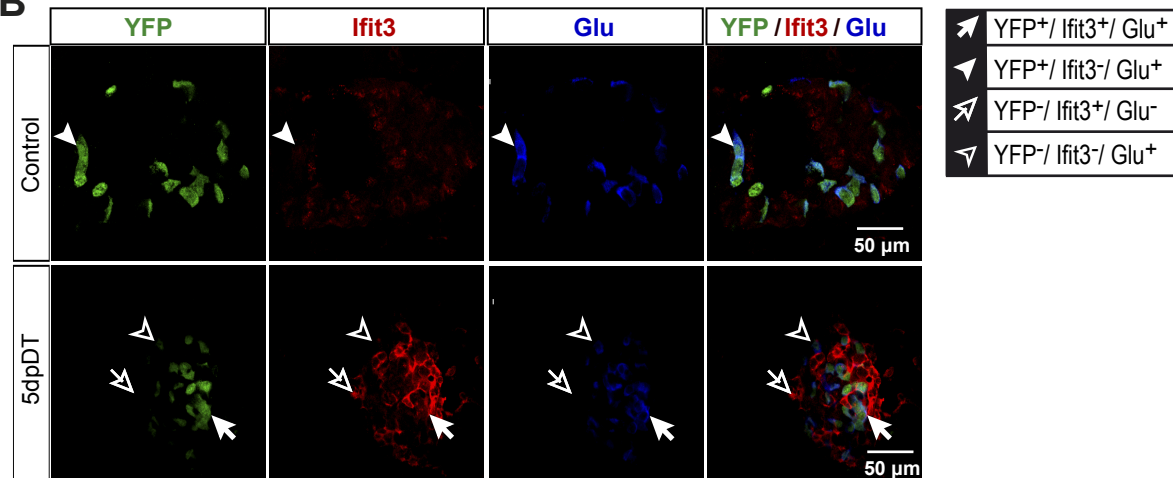

Supplement: Supplementary file 1 — Additional file 1: Figure S1. Most insulin+ cells are ablated from 15dpDT and on. (A) Mean blood glucose levels for the mice used for the 5, 15 and 30 dpDT RNA-seq experiments. (B) Representative immunofluorescence staining of pancreas sections obtained from Control, 5dpDT and 15dpDT treated mice. (C) Percentage of cells expressing Insulin (Ins+) and YFP, expressed relative to the total number of cells in the islets. Total number of islet cells (expressed as mean ± SEM) counted per replicate: Control (784 ± 71, 3 biological replicates), 5dpDT (442 ± 111, 3 biological replicates), 15dpDT (301 ± 168, 2 biological replicates). Figure S2. Signaling pathways modulated in α-cells after acute β-cell loss. (A) Heatmap showing the Normalized Enrichment Score (NES) for selected Gene Set Enrichment Analysis (GSEA) results. T1: Ctrl vs 5dpDT α-cells, T2: 5dpDT vs 15dpDT α-cells, T3: 15dpDT vs 30dpDT α-cells. Genes with higher expression in Ctrl, relative to 5dpDT α-cells (i.e. enriched in Ctrl, with a positive NES), are associated with Smoothened signaling. Genes downregulated in 15dpDT, relative to 5dpDT α-cells (i.e. enriched in 5dpDT, with a positive NES), are associated with MET, mTOR and Rac1 signaling (among other pathways shown). Several of the pathways downregulated in T2 are upregulated in 30dpDT, relative to 15dpDT α-cells (see main text discussion for further details). All results presented are significant considering a P-value < 0.05 and FDR < 0.25. (B) Schematic summarizing the stepwise up- and downregulation of signaling pathways in α-cells as they transition from homeostasis (Ctrl α-cells) to 30dpDT. Figure S3. Epigenomic analysis reveals candidate genes and regulatory elements driving the α-cell response to β-cell loss. (A) Integrative epigenomic maps of the loci of selected cluster 1–5 marker genes showing human islet ChIP-seq signal for 5 key pancreatic islet transcription factors, histone modification enrichment profiles associated with active (H3K27ac) promo [file 12864_2021_7812_MOESM1_ESM.zip › Figure_S5.pdf]

**A**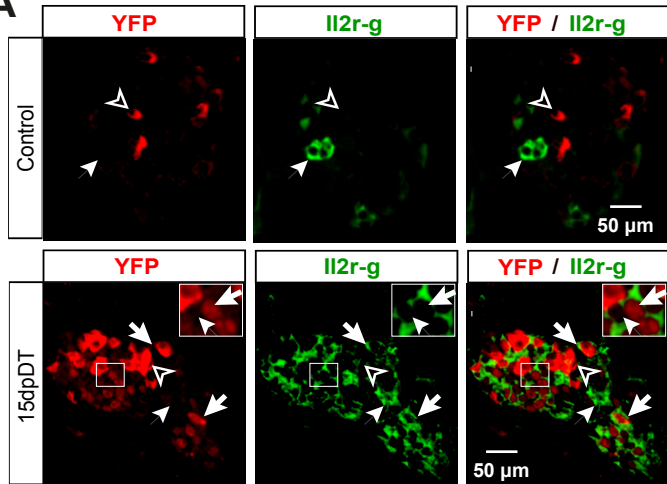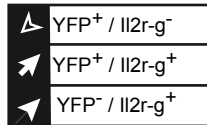**B**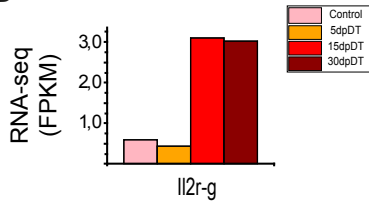

Supplement: Supplementary file 1 — Additional file 1: Figure S1. Most insulin+ cells are ablated from 15dpDT and on. (A) Mean blood glucose levels for the mice used for the 5, 15 and 30 dpDT RNA-seq experiments. (B) Representative immunofluorescence staining of pancreas sections obtained from Control, 5dpDT and 15dpDT treated mice. (C) Percentage of cells expressing Insulin (Ins+) and YFP, expressed relative to the total number of cells in the islets. Total number of islet cells (expressed as mean ± SEM) counted per replicate: Control (784 ± 71, 3 biological replicates), 5dpDT (442 ± 111, 3 biological replicates), 15dpDT (301 ± 168, 2 biological replicates). Figure S2. Signaling pathways modulated in α-cells after acute β-cell loss. (A) Heatmap showing the Normalized Enrichment Score (NES) for selected Gene Set Enrichment Analysis (GSEA) results. T1: Ctrl vs 5dpDT α-cells, T2: 5dpDT vs 15dpDT α-cells, T3: 15dpDT vs 30dpDT α-cells. Genes with higher expression in Ctrl, relative to 5dpDT α-cells (i.e. enriched in Ctrl, with a positive NES), are associated with Smoothened signaling. Genes downregulated in 15dpDT, relative to 5dpDT α-cells (i.e. enriched in 5dpDT, with a positive NES), are associated with MET, mTOR and Rac1 signaling (among other pathways shown). Several of the pathways downregulated in T2 are upregulated in 30dpDT, relative to 15dpDT α-cells (see main text discussion for further details). All results presented are significant considering a P-value < 0.05 and FDR < 0.25. (B) Schematic summarizing the stepwise up- and downregulation of signaling pathways in α-cells as they transition from homeostasis (Ctrl α-cells) to 30dpDT. Figure S3. Epigenomic analysis reveals candidate genes and regulatory elements driving the α-cell response to β-cell loss. (A) Integrative epigenomic maps of the loci of selected cluster 1–5 marker genes showing human islet ChIP-seq signal for 5 key pancreatic islet transcription factors, histone modification enrichment profiles associated with active (H3K27ac) promo [file 12864_2021_7812_MOESM1_ESM.zip › Figure_S6.pdf]
